# Leader behavior impact on intensive care practitioners' burnout

**DOI:** 10.1186/2197-425X-3-S1-A476

**Published:** 2015-10-01

**Authors:** AS Omar, P Sivadasan, MA Khalil, PJ Ostrowski

**Affiliations:** Hamad Medical Corporation, Cardiothoracic Surgery-Heart Hospital, Doha, Qatar; Faculty of Medicine, Beni Suef University, Critical Care Medicine, Beni Suef, Egypt; Faculty of Medicine, Cairo University, Anaesthesiology, Cairo, Egypt; University of Toronto at Scarborough, Management Department, Scarborough, ON Canada

## Introduction

Global spreads of burnout among healthcare practitioners, particularly within intensive care units (ICUs), has been described as a growing crisis with a variety of unwanted consequences as drawbacks [[1]].

## Objectives

Our primary objective was to explore the prevalence of burnout in this area among different healthcare givers; we also focused on identifying the contributing factors as well as the role of empowerment and leadership impact.

## Methods

We employed a cross-sectional descriptive study with purposive sampling. A combined methodological approach (quantitative and qualitative) was used with questionnaires. We used five instrument: Conditions of work effectiveness scale (CWES), Work stress scale (WSS), Maslasch Burnout scale (MBI-HSS), Leadership scale (LS), and Empowerment scale (ES).

## Results

We studied 200 healthcare practitioners within medical and surgical ICUs. The case study that focused on Qatari intensive care confirmed a high prevalence of burnout (25.5%), where physicians, nurses, and respiratory therapists were equally at risk (p = 0.19). Younger individuals were more likely to burn out (p = 0.000). We report a high association of burnout with the instruments that we used. Both positive leadership and empowerment had a negative effect on burnout variance (12.4 and 3.8%, respectively) when considering practitioner burnout.

## Conclusions

The reported high burnout rate among practitioners in ICU settings necessitates special attention in terms of positive leadership attitudes; empowerment could serve as an ameliorating factor.

## Grant Acknowledgment

We thank all of the members of the cardiothoracic surgery department and the medical research center of Hamad medical corporation, Qatar and University of Liverpool, UK.

**Table 1:** Burnout relation with other scores and age.

Variable	MBS-HSS (Pearson Correlation)	Significance. (2-tailed)
Age	-0.466** 0.469**	0.000 000
CWES	-0.186**	0.008
WSS	0.469**	0.000
ES	-0.196**	0.006
LS	-0.353**	0.000

*. Correlation is significant at the 0.01 level (2-tailed). MBS-HSS: Maslach Burnout Inventory human services survey; CWES: Condition of work effectiveness scale; WSS: Occupational stress scale; ES: Psychological Empowerment Scale; Leadership Behaviours scale:LS
